# Wheat ear counting in-field conditions: high throughput and low-cost approach using RGB images

**DOI:** 10.1186/s13007-018-0289-4

**Published:** 2018-03-17

**Authors:** Jose A. Fernandez-Gallego, Shawn C. Kefauver, Nieves Aparicio Gutiérrez, María Teresa Nieto-Taladriz, José Luis Araus

**Affiliations:** 1 Plant Physiology Section, Department of Evolutionary Biology, Ecology and Environmental Sciences, Faculty of Biology, University of Barcelona, Diagonal 643, 08028 Barcelona, Spain; 2 Instituto Tecnológico Agrario de Castilla y León (ITACyL), Ctra. Burgos Km. 119, 47071 Valladolid, Spain; 3 Instituto Nacional de Investigación y Tecnología Agraria y Alimentaria (INIA), Ctra. de la Coruña Km. 7.5, 28040 Madrid, Spain

**Keywords:** Digital image processing, Ear counting, Field phenotyping, Laplacian frequency filter, Median filter, Find maxima, Wheat

## Abstract

**Background:**

The number of ears per unit ground area (ear density) is one of the main agronomic yield components in determining grain yield in wheat. A fast evaluation of this attribute may contribute to monitoring the efficiency of crop management practices, to an early prediction of grain yield or as a phenotyping trait in breeding programs. Currently the number of ears is counted manually, which is time consuming. Moreover, there is no single standardized protocol for counting the ears. An automatic ear-counting algorithm is proposed to estimate ear density under field conditions based on zenithal color digital images taken from above the crop in natural light conditions. Field trials were carried out at two sites in Spain during the 2014/2015 crop season on a set of 24 varieties of durum wheat with two growing conditions per site. The algorithm for counting uses three steps: (1) a Laplacian frequency filter chosen to remove low and high frequency elements appearing in an image, (2) a Median filter to reduce high noise still present around the ears and (3) segmentation using *Find Maxima* to segment local peaks and determine the ear count within the image.

**Results:**

The results demonstrate high success rate (higher than 90%) between the algorithm counts and the manual (image-based) ear counts, and precision, with a low standard deviation (around 5%). The relationships between algorithm ear counts and grain yield was also significant and greater than the correlation with manual (field-based) ear counts. In this approach, results demonstrate that automatic ear counting performed on data captured around anthesis correlated better with grain yield than with images captured at later stages when the low performance of ear counting at late grain filling stages was associated with the loss of contrast between canopy and ears.

**Conclusions:**

Developing robust, low-cost and efficient field methods to assess wheat ear density, as a major agronomic component of yield, is highly relevant for phenotyping efforts towards increases in grain yield. Although the phenological stage of measurements is important, the robust image analysis algorithm presented here appears to be amenable from aerial or other automated platforms.

**Electronic supplementary material:**

The online version of this article (10.1186/s13007-018-0289-4) contains supplementary material, which is available to authorized users.

## Background

The number of ears per unit ground area (ear density) is one of the main agronomical components that determines grain yield in wheat and other cereals, together with the number of grains per ear and the thousand kernel weight [1]. Nevertheless, different studies have shown that while the number of grains per unit ground area is usually the best correlated parameter with grain yield, the correlations of other major agronomical components such as ear density or number of grain per ear are weaker, and grain size is usually the least correlated trait when compared to grain yield [2–4]. Dynamic compensation mechanisms among agronomical yield components appear to be the cause for such contrasts in performance. In current studies of wheat crops, ear counting is performed manually (in situ), which takes time and severely limits its use in breeding as a phenotyping trait, in crop management to monitor plant performance, or to predict grain yield. On the other hand, there is no a single protocol for counting wheat ears, which may further increase experimental variability, particularly when results produced with different methodologies are compared. Moreover, some of the methodological approaches for ear counting are based in the use of grain yield and other traits collected at maturity and therefore, they are not amenable for early yield prediction. Automatic image processing techniques may represent an alternative for high throughput evaluation of ear density. For example, the use of thermal images may be considered an alternative for ear counting since the temperature of the ear may often be several degrees hotter than the surrounding canopy [5]. However, two major limitations of this approach include the low resolution and high cost of thermal cameras, which makes this approach unfeasible for aerial platforms and prohibitively expensive. Alternatively, techniques based on red/green/blue (RGB) digital images of wheat crops captured under field conditions have been reported previously. These approaches have mainly used techniques of image data extraction that were related to characteristics of texture, segmentation of color, morphological operators and skeletonization [6–8]. In the case of a recent paper [6] aiming to automatically determine the heading time, authors made use of a fixed observation device on a platform located above ground level and provided with two cameras facing the crop from opposite directions that recorded daily photographs of the crop. In the same sense, an earlier study focused specifically on ear counting in wheat has shown fairly good results [7], but required a large camera platform and a matte black background structure supported by a tripod for the acquisition of controlled digital images. This structure allowed for avoiding excessive light conditions and unwanted image effects produced by sunlight and shadows, but would greatly hinder its practical application under field conditions. Moreover, these previous approaches have been tested on only one single awnless variety of wheat. In similar work done by Liu et al. [8], they developed an algorithm to calculate the wheat ear count using a database of images in RGB color space and different conditions of planting (drilling and broadcasting); however, the performance was not deemed satisfactory [6], most likely because the counting accuracy was calculated using different sections of a single image rather than testing accuracy in the whole image.

Another example is the automatic ear counting algorithm developed at Rothamsted Research (UK) and tested for example on the FieldScanalyzer of Lemnatec Ltd. This automatic ear counting algorithm, based on RGB images, includes edge detection methods, dilating the lines detected and filling the holes and empty regions. It has been used with good accuracy for counting ear density in a panel composed by five awnless wheat varieties growing under different nitrogen conditions [9]. The camera was installed in an automatic system which moves above the crop in a three dimensional space. Besides its huge cost, this platform can only be used at this particular site, the image processing system uses greyscale images, and thus omits potentially useful RGB information, and to date it has been tested mostly on awnless wheat varieties and across a wide range of ear densities generated largely through different nitrogen fertilization levels, which is not representative of typical growing conditions [2, 3].

Other similar automatic counting approaches using high resolution zenithal RGB images have been developed to estimate tree density. For that purpose, different image processing techniques have been used, which are closely related with the algorithm proposed in this work. Tree crown detection through aerial and high resolution satellite images has often employed smoothing filters to simplify crown form and reduce image noise [10–13]. Also, local maxima filters have been applied on high spatial resolution to detect possible tree crown centers [10–14]. In the case of applications aiming at fruit measure and recognition (e.g. apple, blueberry, grape, mango) like systems have used high resolution images in RGB color space, in order to optimize the visual characteristics of the target objects, followed by segmentation process tasks [15–17]. Alternatively, different color space transformations have been used [18–23]. In most cases, the use of regular digital cameras have been proposed [24–28] due to their high resolution, cost-effectiveness, speed and reliability.

This work proposes a simple system for the automatic quantification of ear density under field conditions based on images acquired by conventional digital cameras. The system uses natural light conditions and therefore is simple to use and may be adaptable to work from aerial platforms. In our study, zenithal images were taken by holding an RGB camera by hand above the crop. Ears per square meter units are calculated using the camera specifications, lens focal length and the distance between the canopy and the camera [29]. First, we applied a Laplacian frequency enhancement to remove part of the soil, leaves and unwanted brightness from the image. Then, similar to other previous automatic image enhancement, segmentation and counting approaches, we employed a median filter as a smoothing technique to further reduce high frequency noise and finally local maximums to determine local peaks within the image for the purpose of wheat ear counting. We also tested this ear counting system on simulated greyscale and reduced resolution images using the same data.

## Methods

### Plant material and growth conditions

Field trials were carried out, during the 2014/2015 crop season at the experimental stations of Colmenar de Oreja (40°04′N, 3°31′W) near Aranjuez and Zamadueñas (41°42′N, 4°42′W) near Valladolid belonging to the Instituto Nacional de Investigación y Tecnología Agraria y Alimentaria (INIA) of Spain and to the Instituto de Tecnología Agraria de Castilla y León (ITACyL), respectively. The average annual precipitation corresponding to Aranjuez area is about 425 mm and the average annual temperature is 13.7 °C, whereas in the case of Valladolid annual averages are 386.2 mm and 11.6 °C. In the case of the Aranjuez trials, the field was fertilized before planting with 400 kg ha^−1^ of a 15:15:15 N:P:K (15% N, 15% P_2_O_5_, 15% K_2_O) fertilizer. A second application of 150 kg ha^−1^ of urea 46% dilution was applied before stem elongation. For the Valladolid trials the field was fertilized before planting with 300 kg ha^−1^ of a 8:15:15 N:P:K (8% N, 15% P_2_O_5_, 15% K_2_O) fertilizer and a second application of 300 NAC-fertilizer kg ha^−1^ was applied before stem elongation.

Twenty-four durum wheat cultivars (*Triticum turgidum* L. subsp. *durum* (Desf) Husn.) post Green Revolution and cultivated in Spain during the past four decades were grown (cvs Amilcar, Avispa, Bólido, Bolo, Burgos, Claudio, Don Ricardo, Don Pedro, Dorondón, Don Sebastian, Gallareta, Iride, Kiko Nick, Mexa, Pelayo, Ramírez, Pelayo, Simeto, Sula Olivadur, Tussur, Martinur, Scupur and Vitrón), all of which had awns. For each site, two growing conditions were assayed: rainfed and supplemental irrigation. For each growing condition, the experimental design was established in randomized blocks with three replicates and a total of 72 plots. Planting took place on November 21, 2014 and November 24, 2014, for Aranjuez and Valladolid, respectively, with a planting density of 250 seeds per square meter. The plots had an area of 7 × 1.5 m^2^ with a spacing distance of 0.2 m between rows. Rainfall during the 2014/2015 crop season was 206 mm and 257 mm and the average temperature was 11.3 and 10.3 °C for Aranjuez and Valladolid, respectively. For the trial under supplemental irrigation, six irrigations were provided at both sites from stem elongation to around 2 weeks after anthesis, totaling 125 mm of water. Harvest was carried out on July 20, 2015 and July 22, 2015, for Aranjuez and Valladolid, respectively and then grain yield was evaluated. In addition, the number of ears per united of grown area (ear density) was measured manually using different approaches. In Aranjuez it was calculated from the total grain yield divided by the weight of kernels per ear. To that end 10 ears per plot were sampled at maturity, threshed and the total kernel weight per spike ear measured. In the case of Valladolid, the total number of ears was counted in two half-a-meter row sections per plot and then the number of ears per united area calculated.

### RGB images

For each plot, one digital RGB picture was taken under natural light conditions by holding the camera at approximately 1.0 m above the plant canopy, in a zenithal plane and focusing near the center of each plot. The images from the first and third visits were acquired with an Olympus E-M10, 16-megapixel resolution camera with a 4/3″ sensor using a 14 mm lens, triggered at a speed of 1/125 s with the aperture programmed in automatic mode. For the second date of measurement, the images were acquired with an Olympus DZ-105, 16-megapixel resolution camera with a 1/2.3″ sensor using a 35 mm lens, triggered at a speed of 1/250 s with the aperture programmed in automatic mode. All images had a native resolution of 4608 × 3456 pixels and were stored in JPG format using the sRGB color standard [30].

**Fig. 1:**
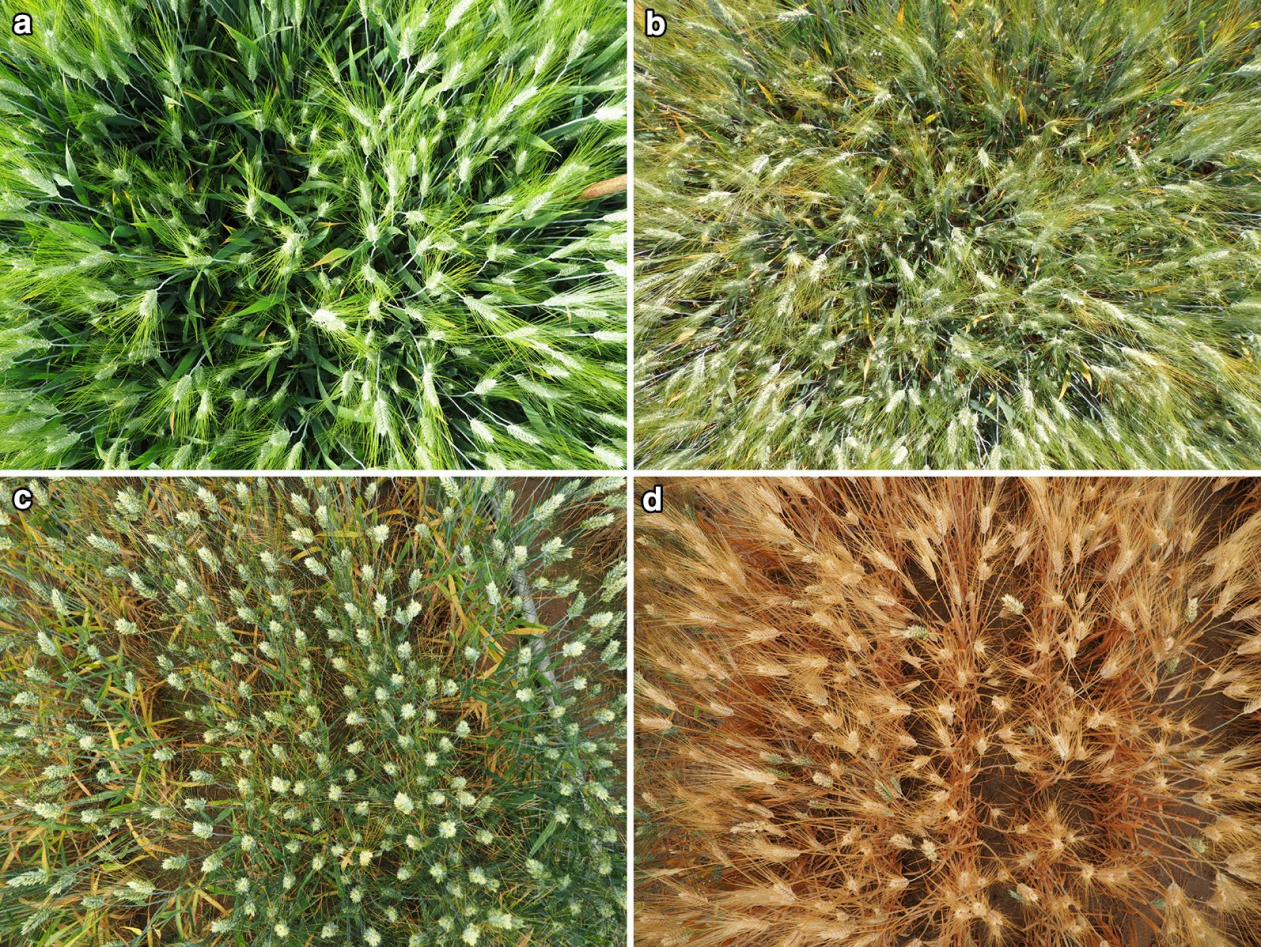
Images of plots at different stages of growth and treatments (Image Database). **a** Aranjuez Irrigated (*first measurement*) cv Martinur, **b** Aranjuez Rainfed (*second measurement*) cv Martinur, **c** Valladolid Irrigated (*third measurement*) cv Amilcar, **d** Valladolid Rainfed (*third measurement*) cv Amilcar

Measurements were performed at three dates: May 12, May 25 and June 8, for both rainfed and support irrigation trials at Aranjuez, and May 14, May 28 and June 9, 2015 for both trials at Valladolid, coincident with the development stages of anthesis (first measurement), middle grain filling (second measurement) and late grain filling (third measurement), respectively, thereby, the colors within the scene changed and depended on not only on the wheat variety but also on its growth stage (Fig. 1). The calculated ear densities (ears/m^2^) remained constant for each date of measurement as the same camera and specification were used for each field visit. The normalized difference vegetation index (NDVI) was measured using a portable spectroradiometer (GreenSeeker handheld crop sensor, Trimble, USA) for the same plots and on the same dates as the RGB image captures.

Preliminary evaluations discarded all images taken late in the afternoon due to the shadows created inside the canopy by the low angle of the incident sunlight, such that all pictures used for further analysis were acquired within 2 h of solar noon or diffuse light conditions. The Additional file 1: Fig. S1 shows images of plots taken under different incident sunlight conditions and growth stages for crops grown under different water regimes. The image (A) was acquired under direct sunlight within 2 h of solar noon, and (B) and (C) were acquired under diffuse light conditions. The ears in (A) and (B) remain contrasted between ears, leaves, and soil due to the irrigated treatment. The ears in (C) are not contrasted due to the change (yellowing) in canopy color by the later stage of growth (earlier senescence) of the rainfed treatment. Although (C) was taken under optimal sunlight conditions, the stage of growth is not appropriate because of the lack of contrast; this was considered as not an optimal phenological condition. As such, both (A) and (B) are considered as taken under optimal light and/or phenological conditions. On the other hand, the ears in (D) are poorly contrasted even though the growth stage was considered appropriate, as the image was taken late in the afternoon; we had to discard these images due to shadows and brightness created inside the canopy by the low angle of the incident sunlight.

### Automatic ear-counting system

**Fig. 2:**
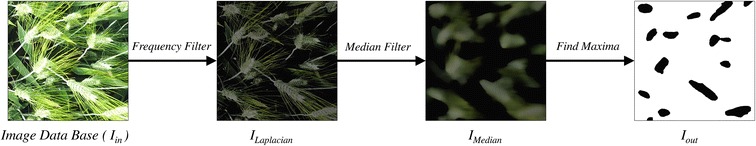
Image processing proposed steps: (i) Laplacian frequency filter (ii) Median filter (iii) *Find Maxima*

The pipeline algorithm for counting consists of three steps: (1) Laplacian frequency filter, (2) Median filter, and (3) segmentation using *Find Maxima*. Greyscale and reduced resolution image simulations were conducted by applying the image conversion prior to the first step. As first step of the image processing system we have chosen a Laplacian filter, due to the wide frequency range of the elements (such as awns, leaves, soil and others unwanted objects) appearing in an image. The second step uses a median spatial filter to reduce the high frequency noise still present around the ears. Finally, we apply the *Find Maxima* segmentation technique, where ears detection was determined by local peaks found within the image. The output of the system is the binary image *I*_*out*_ (Fig. 2). The algorithm was developed in ImageJ software [31].

The Laplacian filter has been used with the aim of detecting changes in the different directions of the image by using a second-order derivative filter [32]. Implementation of the filter was done using *ImageJ* and an extension with a Laplacian filter [33]. This isotropic filter performs as a high frequency enhancement [34] and responds independently of the discontinuities within the image [35]. Equation (1) shows the mathematical frequency model of the Laplacian filter.

1 $$H(u,v) = -\, 4\pi^{2} \left[ {\left( {u - \frac{M}{2}} \right)^{2} + \left( {v + \frac{N}{2}} \right)^{2} } \right],$$

where *H*(*u,v*) represents the transfer function of the Laplacian filter in the frequency domain. The variables *u* and *v* define the frequency axis and the variables *M* and *N* are the shifter constants (*M/*2*, N/*2) from the origin (0,0), as the result of working with a centered spectrum. The constant − 4*π*^*2*^ is obtained in the Laplacian filter mathematical calculations. The Laplacian enhancement in the frequency domain was used applying Eq. (2).

2 $$I_{Laplacian} (x,y) = {\mathcal{F}}^{ - 1} \left\{ {\left[ {1 - H(u,v)} \right]*{\mathcal{F}}(I_{in} (x,y))} \right\},$$

where *I*_*in*_(*x,y*) is the input image, ${\mathcal{F}}$ represents the Fourier transform and [1 − *H*(*u,v*)] denotes the Laplacian frequency enhancement. The resulting image is saved in *I*_*Laplacian*_(*x,y*). In reference to this stage, Fig. 2 shows the initial image *I*_*in*_ and the output image *I*_*Laplacian*_ where Eq. (2) was used. Using this type of filter, the high frequency information is controlled and appends the image filtered with the original image as a background. This enables the removal of part of the soil, leaves and unwanted brightness in the image of the crop as part of the background elements of the image (Fig. 1).

Further, to reduce the high frequency noise in the image and decrease the influence of the awns and leaves, we employed a median filter. The Median filter uses the values in the neighboring cells and sets up a moving window array to calculate the statistical median function of that array; the result is the new pixel in the output image (*I*_*median*_), as seen in Fig. 2. The Median filter results in the visual effect of smoothing the image [36]. This effect depends of the size of window used, with larger sizes producing a greater smoothing effect. This step used a window size of 64 × 64 pixels to prevent removing the small ears. Equation (3) shows a representation of the spatial filter applied to *I*_*Laplacian*_ who represents the image filtering in the frequency domain. The output of this step is *I*_*median*_.

3 $$I_{median} = median\left( {I_{Laplacian} } \right)$$

This filter guarantees as output an image with same pixel values as from the input image and contributes to the reduction of high frequency noise [37]. In the final stage of image processing, the local maximums were detected using the *Find Maxima* algorithm implemented in *ImageJ* [38]. The technique determines local peaks within the image; in that way, it finds the ears because, after filtering, each peak in the image represents a wheat ear. The algorithm creates a binary image segmentation using the pixel value from each local maxima and the nearest neighbor pixel variance to identify the wheat ear shapes in the filtered image, with the white pixels indicating soil, leaves or awns; and the black pixels indicating wheat ears detected in the image.

**Fig. 3:**
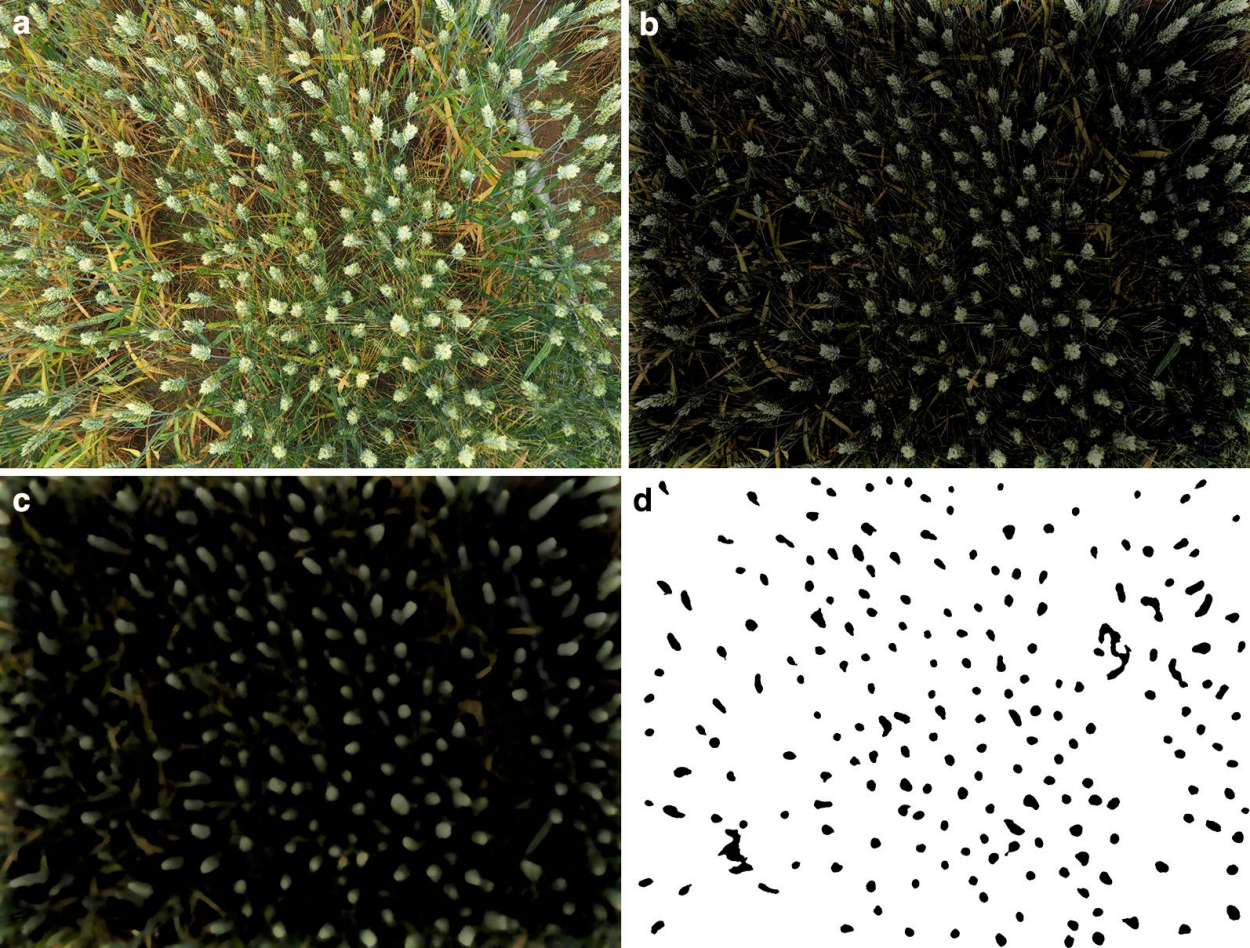
Image processing system using image with completed size. **a** Input image, **b** Laplacian filter, **c** Median filter, **d** *Find Maxima* (*I*_*out*_)

Figure 3 shows the stages of image processing systems using a full-size image. The output image (*I*_*out*_) is used for counting the number of ears in each scene. Each isolated region in black color is considered an ear. The number of regions in *I*_*out*_ image is counted using *Analyzed Particles* implemented in *ImageJ* [38].

### Algorithm validation

The performance of the image processing system for to automatically counting the ears appearing in an image was tested in the images taken from anthesis to maturity (Fig. 1). In order to validate the algorithm, the output of *I*_*out*_ was compared with the manual image-based ear counting on the same image. *I*_*out*_ in Fig. 2, depicts the binary image where the connected pixels in black color are considered like a wheat ear automatically detected by the image processing system; each of these regions are added and the final result is referred to as the *algorithm counting*. Besides, the number of ears in a subset of images has been counted manually (ground truth) and is referred to as the *manual counting*. For Aranjuez, the validation-data manual counting included 72 images, corresponding to the irrigated trial and 24 images belonging to the first block of the rainfed trial from the *first measurement*. For Valladolid, the validation database included 24 ground truth images, taken at the third data measurement, corresponding to the first block of the irrigation and rainfed trials, respectively. For the manual counting we manually marked each ear in the original image and then the number of marks in the image were counted using a simple algorithm developed for counting the number of marks manually selected within the image. In order to determine the success of the automatic ear-counting algorithm, we employed the percentage of error. The success rate in percentage was obtained as the difference between 100% and the relative difference between the manual counting and the algorithm counting (expressed as the difference in the absolute values of the manual and automatic counting, divided by manual counting and multiplied by 100%). We have converted the corresponding image ear-counting numbers in terms of ears per square meter in order to correlate these values with grain yield using the standard agronomical units.

### Statistical analysis

Data was analyzed using InfoStat version 2014 ([39], www.infostat.com.ar) from the National University of Córdoba, Argentina. Pearson correlation coefficients and linear regression were used to analyze the relationship between automatic and manual counting and compare the automatic counting against grain yield. The data was plotted using SigmaPlot version 12 (Systat Software, Inc., San Jose California USA).

## Results

### Success rate and linear regression between the algorithm and manual counts

The success rate in the number of detected ears using the ground truth (manual counting) compared with the automatic counting derived from the image processing was calculated (Table 1).

**Table 1:** Percentage of success of the automatic counting at the original RGB resolution, greyscale and the resized imagery validation results

Trial, date of sampling		Original RGB	Greyscale	×1/2	×1/4	×1/8	×1/16	×1/32
Aranjuez, May 12	μ	92.39%	88.52%	92.14%	91.6%	88.98%	81.10%	62.94%
Irrigated	σ	6.23	9.90	5.89	6.04	7.06	8.75	7.51
72 images	r	0.79	0.73	0.78	0.78	0.76	0.71	0.64
Aranjuez. May 12	μ	91.06%	90.78%	90.30%	89.25%	85.50%	77.41%	60.12%
Irrigated	σ	6.37	8.99	6.29	6.79	7.48	8.66	6.89
24 images	r	0.78	0.76	0.77	0.79	0.77	0.76	0.74
Aranjuez. May 12	μ	91.70%	93.01%	91.15%	89.41%	84.92%	76.59%	59.59%
Rainfed	σ	6.96	4.57	7.79	8.7	9.37	8.82	7.60
24 images	r	0.72	0.80	0.69	0.67	0.65	0.62	0.47
Valladolid. June 9	μ	89.79%	80.56%	89.22%	87.67%	82.47%	72.47%	50.97%
Irrigated	σ	10.14	12.19	10.52	11.07	12.47	15.32	14.09
24 images	r	0.87	0.87	0.87	0.86	0.824	0.80	0.73
Valladolid. June 9	μ	31.86%	65.36%	31.12%	29.64%	27.01%	22.65%	14.02%
Rainfed	σ	7.54	11.53	7.38	7.02	6.51	5.8	4.32
24 images	r	0.42	0.35	0.39	0.38	0.39	0.34	0.34

Different sites and phenological stages across the set of 24 durum wheat varieties were assayed. Values presented are the means of percentage of success (μ), standard deviation (σ) and Pearson correlation coefficient (r)

**Fig. 4:**
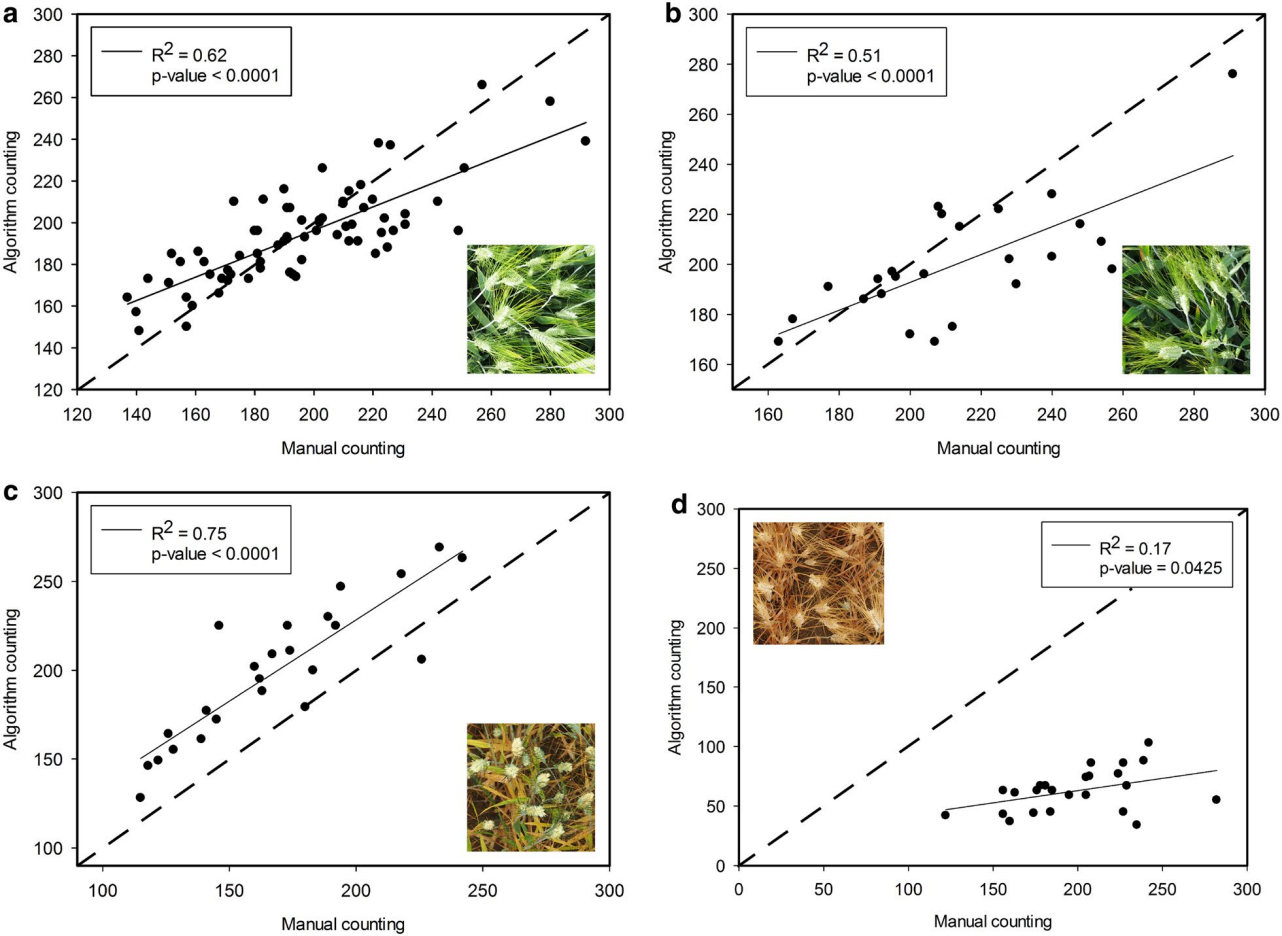
Plots of Manual counting versus Algorithm counting at different growth stages. 72 plots: **a** Aranjuez Irrigated May 12. 24 plots: **b** Aranjuez Rainfed May 12. **c** Valladolid Irrigated June 9. **d** Valladolid Rainfed June 9

Furthermore, the linear regression between the manual counting and the algorithm counting was calculated for the 72 irrigated and 24 rainfed plots from Aranjuez at anthesis (first sampling date) as well as across 24 plots from the irrigated and another 24 plots from the rainfed trials of Valladolid at leaf grain filling (Fig. 4).

The success rate demonstrated the high accuracy of the algorithm counting with regard the manual ear counting and the standard deviation values imply a small data dispersion (Table 1). Thus, mean and standard deviation values for Aranjuez derived from the images taken at anthesis (mid-May) in the irrigated trial exhibited robust accuracy when applied to only one replicate (μ = 91.06%, σ = 6.37) or to the three replicates of each genotype (μ = 92.39, σ = 6.23). Performance for the rainfed trial at Aranjuez during the first measurement data was similar (μ = 91.71%, σ = 6.96). Performance was almost as strong (μ = 89.79%, σ = 10.14) at the irrigated Valladolid trial measured at late grain filling (third valuation, early June). However the performance in the rainfed trial of Valladolid measured on early June was much lower (μ = 31.86%, σ = 7.54), suggesting the growth stage affect the correct identification of ears. In the same sense the relationships between the manual and algorithm ear counting for Aranjuez at mid-May (*irrigated* R^2^ = 0.62; *rainfed* R^2^ = 0.51) and irrigated Valladolid at late grain filling (R^2^ = 0.75) were positive and strong (Fig. 4), with the irrigated Valladolid late grain filling additionally demonstrating a close 1:1 relationship. In the case of rainfed Valladolid at late grain filling the correlation, even if significant, was weaker (R^2^ = 0.17) and much further from a 1:1 slope that the rest.

### Simulating greyscale and lower resolution imagery

The performance of the algorithm was further tested through the simulation of images in greyscale and at lower resolutions using the original high resolution image data. The greyscale images were converted by averaging the RGB color bands. The lower resolution images were resized to five different resolutions by dividing the original image size by two (obtaining an image of 2304 × 1729 pixels) as far as obtain the smallest image size dividing by 32 (114 × 108 pixels). The images were resized using average pixel values, with no interpolation techniques applied. We have used the same algorithm pipeline proposed for greyscale and lower resolution, although in the Median filter step (Eq. 3), we reduced the moving window size in proportion to the image resizing to match the subsequent size of the wheat ears in the reduced image. Manual image-based counting was used as the validation data as before. Table 1 gives the statistical summary results obtained for Aranjuez and Valladolid plots. Additional file 2: Fig. S2 shows the resized imagery simulation; the images were resized using average pixel values, with no interpolation of values.

The greyscale results show, with respect to the original RGB images, a decrease in up to 9.23% in success rate while maintaining a similar correlation as the RGB results in the irrigated trials. While the greyscale resulted in an increase in success rate for the Valladolid rainfed trial, little changes were observed in correlation and success rate for the Aranjuez rainfed trial.

The lower resolution results show a decrease of < 1% in success rate when the images were reduced to a half of its original size. Success rates decreased by a maximum of 2.29, 7.32 and 17.32% for image size divided by four, eight and 16 values, respectively. For the smallest image size, success rate decreases as much as 38.82%. Standard deviation values exhibited robust accuracy at moderately lower resolutions and Pearson correlation coefficients remained close to original values, for all but the smallest simulated image size where the correlation values shifted markedly from the original values.

### Understanding algorithm errors

**Fig. 5:**
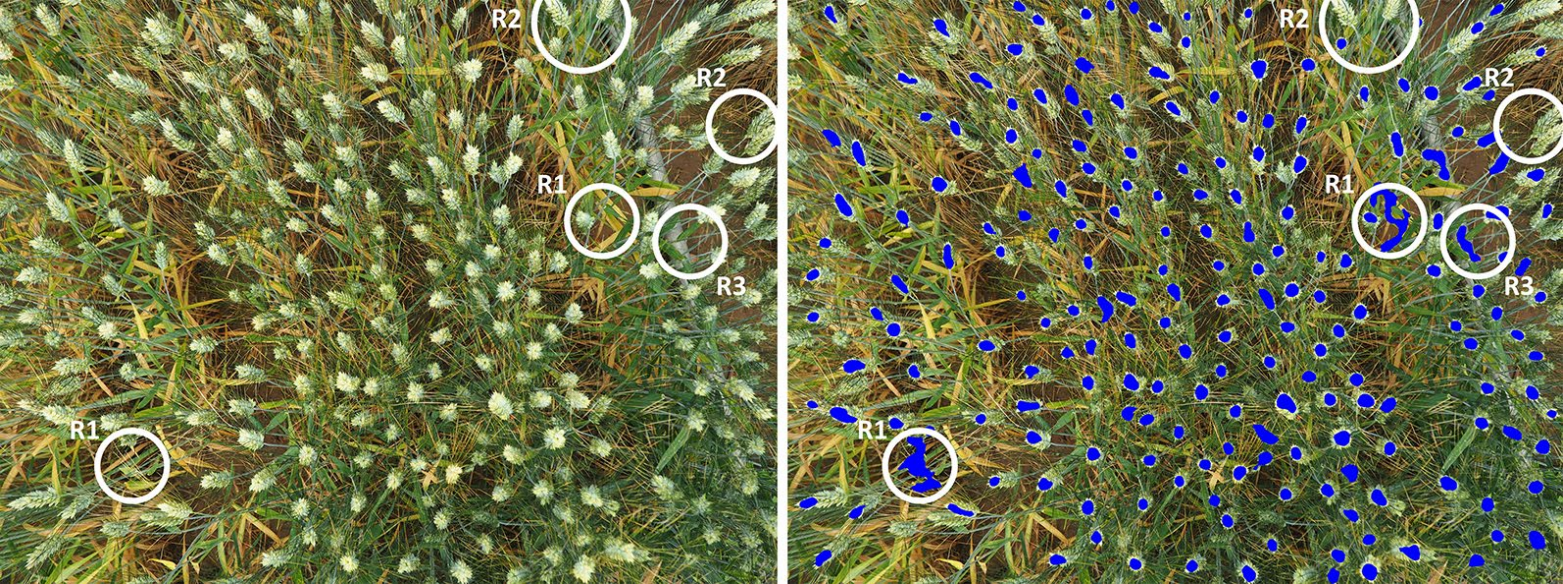
Algorithm error regions. *I*_*in*_ and *I*_*out*_ images. Blue marks in the *I*_*out*_ indicate algorithm results

Figure 5 shows the input and output images, with each blue pixels representing an ear automatically detected by the proposed algorithm. There are three regions in the image indicating examples where the algorithm has not worked properly. For example, region *R1* shows false positives where pixels labeled as ear actually corresponded to leaves and resulted in irregularities in the ear counting. As a result, pixels from leaves were united with ear pixels in the *Find Maxima* step and included together as one combined area. In region *R2*, false negatives resulted in ears that were not detected by the algorithm because the contrast between the ear and soil was not great enough and the segmentation algorithm discarded that region. In case of region *R3*, whereas the algorithm labeled the area as an ear, those pixels are soil and noise being a result of background brightness caused by a foreign object.

### Relationship between algorithm counting and grain yield

The relationship of grain yield against the ear counting calculated with the algorithm in terms of ears per square meter (ears/m^2^) at the three measurement dates as well as the manual in situ counting values were assessed. In Table 2 we provide a statistical summary of the results obtained with the complete dataset of plots from the rainfed and support-irrigation trials of the two experimental sites (288 plots), as well as within each trial and experimental site (72 plots). Moreover, NDVI is included as a standard indicator of crop greenness and vigor.

**Table 2:** Statistical results of the relationships across the whole set of plots (288), as well as across the set of plots of each trial (72) between grain yield and the ear counting using the algorithm (ears/m^2^) in the first, second and third date of measurement as well as the manual in situ counting

Determination coefficient (R^2^), Pearson correlation (r) and mean (μ) ± standard deviation for NDVI	First measurement	Second measurement	Third measurement	Manual in situ counting
Whole dataset (288)	R^2^ = 0.30***	R^2^ = 0.08***	R^2^ = 0.05***	R^2^ = 0.24***
	r = 0.55***	r = 0.28***	r = 0.21***	r = 0.49***
Aranjuez Irrigated Dataset (72)	R^2^ = 0.05^ns^	R^2^ = 0.05^ns^	R^2^ = 0.02^ns^	R^2^ = 0.18**
	r = 0.22^ns^	r = -0.04^ns^	r = 0.14^ns^	r = 0.43**
	μ = 0.78 ± 0.03	μ = 0.71 ± 0.07	μ = 0.29 ± 0.14	
Aranjuez Rainfed Dataset (72)	R^2^ = 0.05^ns^	R^2^ = 0.02^ns^	R^2^ = 0.02^ns^	R^2^ = 0.53***
	r = 0.22^ns^	r = 0.16^ns^	r = 0.14^ns^	r = 0.73***
	μ = 0.76 ± 0.02	μ = 0.67 ± 0.04	μ = 0.17 ± 0.11	
Valladolid Irrigated Dataset (72)	R^2^ = 0.06*	R^2^ = 0.0049^ns^	R^2^ = 0.06*	R^2^ = 0.01^ns^
	r = − 0.24*	r = − 0.07^ns^	r = 0.25*	r = 0.07^ns^
	μ = 0.73 ± 0.03	μ = 0.67 ± 0.05	μ = 0.45 ± 0.10	
Valladolid Rainfed Dataset (72)	R^2^ = 0.07*	R^2^ = 0.05^ns^	R^2^ = 0.18***	R^2^ = 0.05*
	r = 0.26*	r = 0.22^ns^	r = 0.43***	r = 0.23*
	μ = 0.66 ± 0.04	μ = 0.41 ± 0.05	μ = 0.18 ± 0.14	

The mean (μ) ± standard deviation of the normalized difference vegetation index (NDVI) values, across the whole set of plots within each trial is also included for reference

*ns* no significant

*p value < 0.05; **p value < 0.01; ***p value < 0.001

**Fig. 6:**
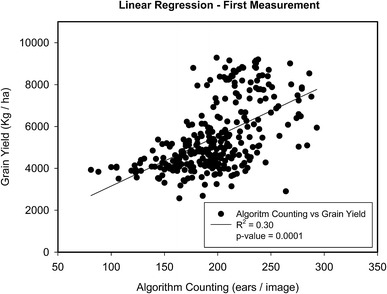
Fitting regression of the grain yield against the ear counting, estimated during the first measurement and for the whole dataset (288 plots) using the algorithm counting

The results show that the ear-counting algorithm correlated better with grain yield at the *first measurement date* (R^2^ = 0.30) than at the *second measurement date* (R^2^ = 0.08) or the *third measurement date* (R^2^ = 0.05). The relationship of the manual in situ counting against grain yield was R^2^ = 0.24. The pattern of the relationship between the automatic ear counting and grain yield was in all cases lineal (Fig. 6).

## Discussion

Working in field conditions implies many considerations, especially when plant phenotyping tasks are developed. Ear density is frequently identified as the main agronomical component of yield and it appears to be the most relevant towards future increases in grain yield ([4] and references therein). Developing low-cost, fast and easy-to-implement field methods to assess wheat ear density is therefore critical to developing wheat varieties with greater yield. We propose the use of a simple RGB image acquisition method holding the camera above the crop canopy, whereas the automatic image processing includes robust algorithms designed for different wheat varieties and growth stages. Previous studies in ear recognition have used acquisition methods/structures which include the use of enclosing structure or a fixed camera support [7, 8] or even artificial light [40] that would greatly limit their practical application under field conditions. Moreover, even if good results were achieved, in most cases only a single awnless wheat variety was used. By contrast, our study included 24 wheat varieties with awns of different colors and culms with ears ranging from erect to floppy, which eventually may affect negatively the performance of the algorithm compared to its application on a single awnless variety.

Unlike the use of artificial light and enclosures, together with a camera support (e.g. tripod), our flexible and fast image acquisition technique presents some major challenges related to image processing. Some ears in the image may appear blurred due to plant movements as a result of wind or absolute camera stability. Sharp shadows and bright surfaces may appear in the images as a product of the light conditions (e.g. in a sunny day). The more erect or floppy attitude of the ears may also affect the counting, whereas the presence of awns represents an additional problem since the awns visually overlap with the body of the ear. This is the case of our study performed in durum wheat, a species which always exhibits awns. Finally, changes in the color of the leaves and ears due to crop growth stage, together with differences in crop density and the soil background may also interfere.

As such, in order to provide robust results, the image processing algorithm pipeline must consider different disturbing effects related with shadows, brightness, leaf color, the presence of awns or even overlapping ears. The Laplacian frequency filter contributes to removing or minimizing visual effects from unwanted brightness and background elements, and the Median spatial filter provides an important contribution to smoothing regions and removing noise from regions that still contained high frequency noise because of the presence of awns [33, 36]. Even so, the lower performance of the counting algorithm in the rainfed trial of Valladolid at late grain filling, compared with the results of the irrigated trial at the same date or compared with the trials at anthesis, was most likely due to the differences in color between the first compared with the other three cases. In the case of the rainfed trial of Valladolid at late grain filling, plants were much more senescent compared with the other three cases (Fig. 4). The lack of contrast in the images of the rainfed plots on late grain filling between ears, leaves and soil did not permit consistent ear identification. We consider the stage of growth of this treatment too late for the proposed algorithm. In comparison, the irrigated trial at Valladolid still exhibited sufficient contrast in the upper part of the canopy at the third date of measurements, which contributed to the better performance of the counting algorithm compared to manual counts in terms of precision, r correlation value, and 1:1 relationship between modeled and predicted counts. During grain filling, particularly under Mediterranean conditions, the ears often remain greener longer compared to the leaves and the culm [41], which is essential to provide the necessary contrast between ears, leaves and soil.

In our study, phenotypic correlations across mean values for the 24 genotypes between ear density and grain yield were very poor (in fact absent in most cases) and regardless of whether ear density was directly measured directly in the field or inferred from the automatic counting algorithm. Only in one of the 4 individual trials was manual counting correlated with grain yield, whereas automatic counting was only found to be correlated with grain yield in another trial; in both cases the correlations were found in the rainfed trials (data not shown), explaining in each case about 35% on genotypic variability in grain yield. In fact, compensatory mechanisms between ear density and number of kernels per ear have been reported widely [4, 42], which may account for the lack of correlation between ear density and grain yield in our study. For example, in Valladolid ear density was negatively correlated with the number of grains per ear in both rainfed (r = − 0.31; *p* < 0.01) and irrigated (r = − 0.36; *p* < 0.01) conditions. Additional file 3: Table S1 gives the statistical summary results of mean, standard error, minimum and maximum value for the whole set of four trials as well as within each trial, for grain yield, thousand kernel weight (TKW), number of grains per unit ground area and ear density (number of ears per unit ground area).

Automatic ear counting performed around anthesis correlated better with grain yield than at later stages, when canopy color shifts to yellow. The low performance of ear counting at the late grain filling stages associated with a change (yellowing) in canopy color, including the ears, with the subsequent loss of contrast, may be a reason. An additional factor may be due to the fact ears in the two trials of Aranjuez suffered logging during middle grain filling and strongly increased at late grain filling. In any case, the solid performance of the algorithm at earlier growth stages is viewed as one of its strong points as it may contribute to earlier yield prediction for crop management purposes. In fact, this method may be fully amenable for management purposes since frequently the range of variability in ear density due to environmental causes and agronomical factors (e.g. availability of water, nutrients, other abiotic and biotic factors, planting date and density, etc.) is larger than that due exclusively to genotypic variability. Therefore, the level of precision provided by the method is less critical for crop management than for phenotype selection in breeding programs.

The greyscale image simulations resulted in a decrease in success rate for the irrigated trials, while on the other hand contributing to an improvement in success rate for rainfed trial images, especially for the Valladolid rainfed trial, but the correlation against manual counting for that trial still remained the lowest. This may be a result of the increased senescence of the rainfed trials at the time of data capture, in which the benefits of color contrast between the leaves and the ears had passed, indicating that it was not an optimal moment for data capture. These results therefore suggest that at a specific growth stage the full RGB color information may provide significant improvements over greyscale images.

Further still, in our study, two different models of cameras were used in image acquisition (Olympus E-M10 and DZ-105) and the algorithm was tested for accuracy with greyscale images without color information and at lower resolutions, in order to provide an idea about the possibility of optimizing processing requirements (less computing time with single band images) and using other types of cameras with lower resolution, such mobile phones, action cameras (GoPro), or even similar or higher quality cameras at a greater distance (with or without zoom lens, etc.) such that the same robust algorithm for ear counting may be adaptable to mobile, field or aerial (UAV) phenotyping and precision agriculture platforms.

## Conclusions

This study proposes a low-cost and easy-to implement approach for ear counting. The system uses a handheld camera that easily can be moved across the plots. Moreover, the image analysis algorithm is amenable to other applications, such as early assessment of yield through the acquisition of RGB images from aerial or other automated platforms. Nevertheless, the performance of this image processing system depends on the phenological stage when measurements took place. Mature canopies, with the ears already yellow, are not adequate for ear counting. In our study, different hybrid color spaces were considered as an image pre-processing stage, but there was no difference in input image enhancement or algorithm results. We chose to use the original RGB color space for its benefits in contrast at specific growth stages, although greyscale images can be useful in low color contrast conditions; nevertheless, in future studies it may be interesting to investigate hybrid color spaces or high resolution RGB imagery combined with multispectral or thermal imagery to enhance the performance of the ear counting algorithm.

## Additional files

**Additional file 1. Figure S1.** Images of plots taken under different incident sunlight conditions, and growth stages on crops grown under different water regimes. **A** Image taken at anthesis with direct sunlight within two hours of solar noon in an irrigated plot. **B** Image taken at late grain filling under diffuse light conditions in the morning in an irrigated plot. **C** Image taken during late grain filling under diffuse light conditions near solar noon in a rainfed plot. **D** Image taken late at middle grain filling with direct sunlight in the afternoon.

**Additional file 2. Figure S2.** Resized imagery simulation—input and output images. The images were resided using average pixel values, with no interpolation techniques applied.

**Additional file 3. Table S1.** Values for the whole set of four trials as well as within each trial, for grain yield, thousand kernel weight (TKW), number of grains per unit ground area (grains/m^2^) and ear density (number of ears per unit ground area). For each trait, mean, standard error (SE) and minimum and maximum value across the individual plot.
