# Supplementary material for: Wheat ear counting in-field conditions: high throughput and low-cost approach using RGB images
Source: Plant Methods. 2018 Mar 17;14:22. doi: 10.1186/s13007-018-0289-4 (PMC5857137; doi:10.1186/s13007-018-0289-4)
Supplement: Supplementary file 1 — Additional file 1. Figure S1. Images of plots taken under different incident sunlight conditions, and growth stages on crops grown under different water regimes. A Image taken at anthesis with direct sunlight within two hours of solar noon in an irrigated plot. B Image taken at late grain filling under diffuse light conditions in the morning in an irrigated plot. C Image taken during late grain filling under diffuse light conditions near solar noon in a rainfed plot. D Image taken late at middle grain filling with direct sunlight in the afternoon. [file 13007_2018_289_MOESM1_ESM.docx]

**Wheat ear counting in-field conditions: high throughput and low-cost approach using RGB images**

Jose A. Fernandez-Gallego^a^, Shawn C. Kefauver^a^^*^, Nieves Aparicio Gutiérrez^b^, Maria Teresa Nieto-Taladriz^c^, José Luis Araus^a^


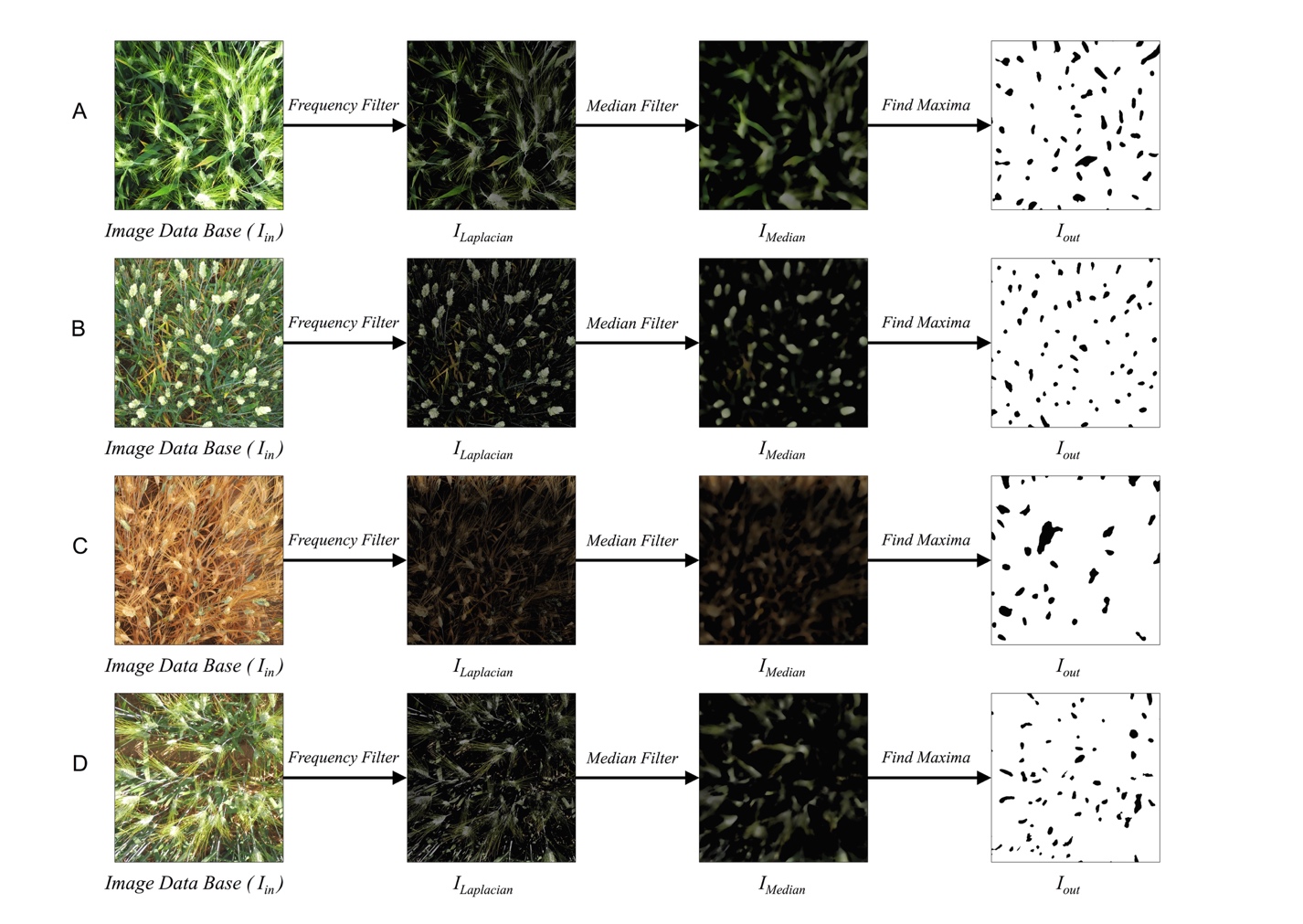


**Figure S1.** Images of plots taken under different incident sunlight conditions, and growth stages on crops grown under different water regimes. A) Image taken at anthesis with direct sunlight within two hours of solar noon in an irrigated plot. B) Image taken at late grain filling under diffuse light conditions in the morning in an irrigated plot. C) Image taken during late grain filling under diffuse light conditions near solar noon in a rainfed plot. D) Image taken late at middle grain filling with direct sunlight in the afternoon
