# Supplementary material for: Wheat ear counting in-field conditions: high throughput and low-cost approach using RGB images
Source: Plant Methods. 2018 Mar 17;14:22. doi: 10.1186/s13007-018-0289-4 (PMC5857137; doi:10.1186/s13007-018-0289-4)
Supplement: Supplementary file 2 — Additional file 2. Figure S2. Resized imagery simulation—input and output images. The images were resided using average pixel values, with no interpolation techniques applied. [file 13007_2018_289_MOESM2_ESM.docx]

**Wheat ear counting in-field conditions: high throughput and low-cost approach using RGB images**

Jose A. Fernandez-Gallego^a^, Shawn C. Kefauver^a^^*^, Nieves Aparicio Gutiérrez^b^, Maria Teresa Nieto-Taladriz^c^, José Luis Araus^a^


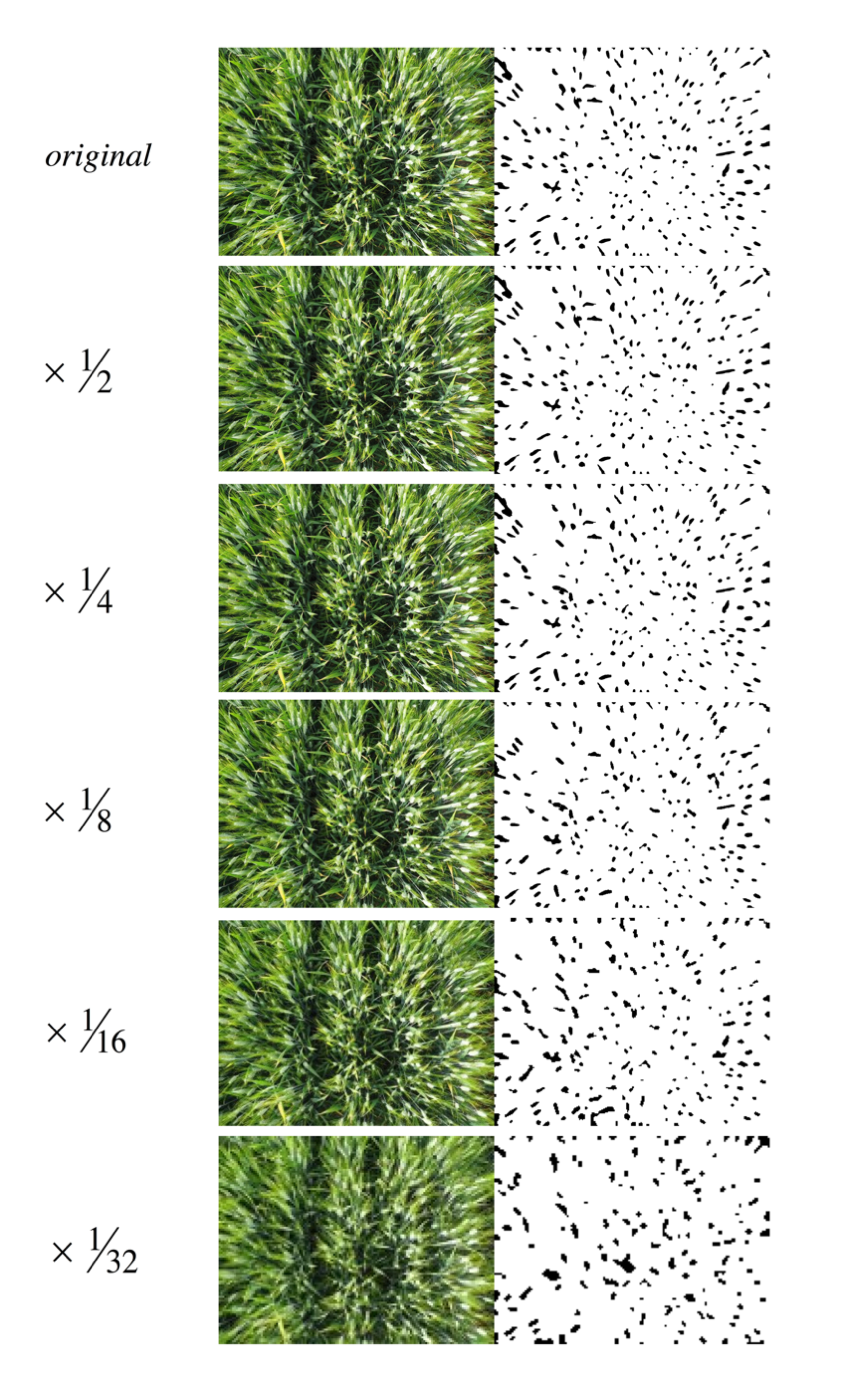


**Figure S2.** Resized imagery simulation – input and output images. The images were resided using average pixel values, with no interpolation techniques applied.
