# Supplementary material for: Wheat ear counting in-field conditions: high throughput and low-cost approach using RGB images
Source: Plant Methods. 2018 Mar 17;14:22. doi: 10.1186/s13007-018-0289-4 (PMC5857137; doi:10.1186/s13007-018-0289-4)
Supplement: Supplementary file 3 — Additional file 3. Table S1. Values for the whole set of four trials as well as within each trial, for grain yield, thousand kernel weight (TKW), number of grains per unit ground area (grains/m2) and ear density (number of ears per unit ground area). For each trait, mean, standard error (SE) and minimum and maximum value across the individual plot. [file 13007_2018_289_MOESM3_ESM.docx]

**Wheat ear counting in-field conditions: high throughput and low-cost approach using RGB images**

Jose A. Fernandez-Gallego^a^, Shawn C. Kefauver^a^^*^, Nieves Aparicio Gutiérrez^b^, Maria Teresa Nieto-Taladriz^c^, José Luis Araus^a^

**Table S1.** Values for the whole set of four trials as well as within each trial, for grain yield, thousand kernel weight (TKW), number of grains per unit ground area (grains/m2) and ear density (number of ears per unit ground area). For each trait, mean, standard error (SE) and minimum and maximum value across the individual plot

| *Trial, database* | *Variable* | *Mean* | *SE* | *Min.* | *Max.* |
| --- | --- | --- | --- | --- | --- |
| *Whole set (288 plots)* | *grain yield [kg/ha]*  *TWK [g]*  *grains/m2*  *ears/m2* | *5499*  *42.3*  *13266*  *410.0* | *97*  *0.5*  *168*  *5.2* | *2220*  *27.9*  *6154*  *205.6* | *9281*  *63.8*  *20361*  *709.0* |
| *Aranjuez Irrigated*  *(72 plots)* | *grain yield [kg/ha]*  *TWK [g]*  *grains/m2*  *ears/m2* | *5126*  *40.3*  *14014*  *487.0* | *88*  *0.5*  *306*  *11.1* | *3245*  *32.3*  *8100*  *313.8* | *6924*  *48.6*  *19663*  *709.0* |
| *Aranjuez Rainfed*  *(72 plots)* | *grain yield [kg/ha]*  *TWK [g]*  *grains/m2*  *ears/m2* | *4648*  *40.8*  *12549*  *405.8* | *104*  *0.5*  *303*  *10.8* | *2220*  *32.0*  *6154*  *205.6* | *6397*  *50.1*  *17846*  *642.5* |
| *Valladolid Irrigated (72 plots)* | *grain yield [kg/ha]*  *TWK [g]*  *grains/m2*  *ears/m2* | *7971*  *52.0*  *14882*  *388.7* | *91*  *0.8*  *347*  *6.3* | *6101*  *33.9*  *9074*  *244.0* | *9281*  *63.8*  *20361*  *524.0* |
| *Valladolid Rainfed (72 plots)* | *grain yield [kg/ha]*  *TWK [g]*  *grains/m2*  *ears/m2* | *4231*  *36.0*  *11618*  *358.3* | *67*  *0.6*  *242*  *5.4* | *2566*  *27.9*  *7718*  *260.0* | *6510*  *47.1*  *16517*  *488.0* |
